# Inhibitory effects of BMP9 on breast cancer cells by regulating their interaction with pre-adipocytes/adipocytes

**DOI:** 10.18632/oncotarget.16271

**Published:** 2017-03-16

**Authors:** Ting Wang, Zhihui Zhang, Ke Wang, Jinshu Wang, Yayun Jiang, Jing Xia, Liyao Gou, Mengyao Liu, Lan Zhou, Tongchuan He, Yan Zhang

**Affiliations:** ^1^ Key Laboratory of Diagnostic Medicine of The Chinese Ministry of Education, School of Clinical Diagnostic and Laboratory Medicine, Chongqing Medical University, Yuzhong District, Chongqing, P.R.China; ^2^ Yongchuan Hospital, Chongqing Medical University, Chongqing, P.R.China; ^3^ Molecular Oncology Laboratory, Department of Surgery, University of Chicago Medical Center, Chicago, IL, USA

**Keywords:** bone morphogenetic protein 9, adipocyte, breast cancer cell, microenvironment, leptin

## Abstract

Bone morphogenetic protein 9 (BMP9) possesses multiple functions, but its effects on breast cancer cells in adipose microenvironment are still unclear. This study aimed to investigate whether BMP9 is able to modulate the interaction between pre-adipocytes/adipocytes and breast cancer cells. An *in vitro* co-culture system was established by using pre-adipocytes/adipocytes and MDA-MB-231 breast cancer cells with BMP9 over-expression. The leptin expression and leptin-induced signaling pathway were evaluated in this co-culture system. MTT assay, EdU assay and flow cytometry were used to assess the proliferation of MDA-MB-231 cells. Wound-healing assay and Transwell migration assay were used to assess the migration of MDA-MB-231 cells. Immunofluorescence staining was used to detect the expression of leptin recepter (ObR) in MDA-MB-231 cells. The expression of key molecules in leptin signaling pathway in co-culture system were detected by Western blotting. MDA-MB-231 cells and pre-adipocytes/adipocytes were inoculated into nude mice, the tumor volume was measured, and the protein expression of key molecules in leptin signaling pathway was detected. Results showed BMP9 inhibited breast tumor growth *in vitro* and *in vivo* and reduced the migration of breast cancer cells *in vitro*. MDA-MB-231 cells with BMP9 over-expression decreased leptin expression in pre-adipocytes/adipocytes and had reduced phosphorylation of STAT3, ERK1/2 and AKT. Taken together, our study indicates that BMP9 can inhibit the growth and metastasis of breast cancer cells, which may be related to interaction between pre-adipocytes/adipocytes and MDA-MB-231 cells via leptin signaling pathway.

## INTRODUCTION

Breast cancer is the most common cancer in women [1]. Although the diagnostic techniques and therapies for breast cancer have been improved in past decades with the development of medicine and technology, cancer relapse, drug resistance, metastasis and other clinical features still lead to a high mortality in breast cancer patients [2]. Studies have shown that obesity has been regarded as an independent risk factor of breast cancer [3, 4]. Increased accumulation of adipose tissue is observed in obese people, and excessive pre-adipocytes and adipocytes lead to the dysregulated secretion of biologically active adipocytokines including leptin [5, 6].

Leptin is a secreted polypeptide with the molecular weight of approximately 16 kD [7, 8]. Several studies have demonstrated the close relationship between obesity and cancer [9–12]. Leptin is able to bind to the leptin receptor (ObR) and then activates the intracellular JAK/STAT, MAPK/ERK1/2 and PI3K/AKT pathways, promoting cancer progression [13]. Over-expression of leptin has been found to promote the proliferation, migration and angiogenesis of breast cancer cell, thereby accelerating the growth and metastasis of breast cancer [11, 14]. Therefore, it is imperative to develop effective strategies to inhibit the tumorigenic effects of leptin for obese patients.

Mounting evidence indicates a close relationship among bone morphogenetic proteins (BMPs), adipocytes and breast cancer [15]. BMPs belong to the transforming growth factor-β (TGF-β) superfamily [16], and were initially identified as osteoinductive cytokines that can promote bone and cartilage formation *in vivo* [17]. Recently, they have been shown to be involved in the regulation of tumor development and bone metastasis [18]. BMP9 (also known as growth differentiation factor 2)is a member of BMPs and has different functions in distinct cancers [19]. For instance, BMP9 can promote the proliferation and migration of liver cancer cells [20], but is able to inhibit the growth of osteosarcoma cells [21]. In a previous study, our results showed that BMP9 inhibited the growth, migration, and invasion of highly malignant MDA-MB-231 and HER2-positive SK-BR-3 cells [22–24], and suppressed the epithelial-mesenchymal transition (EMT) of breast cancer cells in the bone microenvironment [25]. However, the effects of BMP9 on the biobehaviors of breast cancer cells in adipose microenvironment remain unclear. In this study, we investigated the effects of BMP9 on the interaction of breast cancer cells and pre-adipocytes or adipocytes, and explored the potential mechanisms, which may provide evidence for the development of targeted therapy for breast cancer.

## RESULTS

### Effects of pre-adipocytes and adipocytes on the biological behaviors of MDA-MB-231 cells

**Figure 1:**
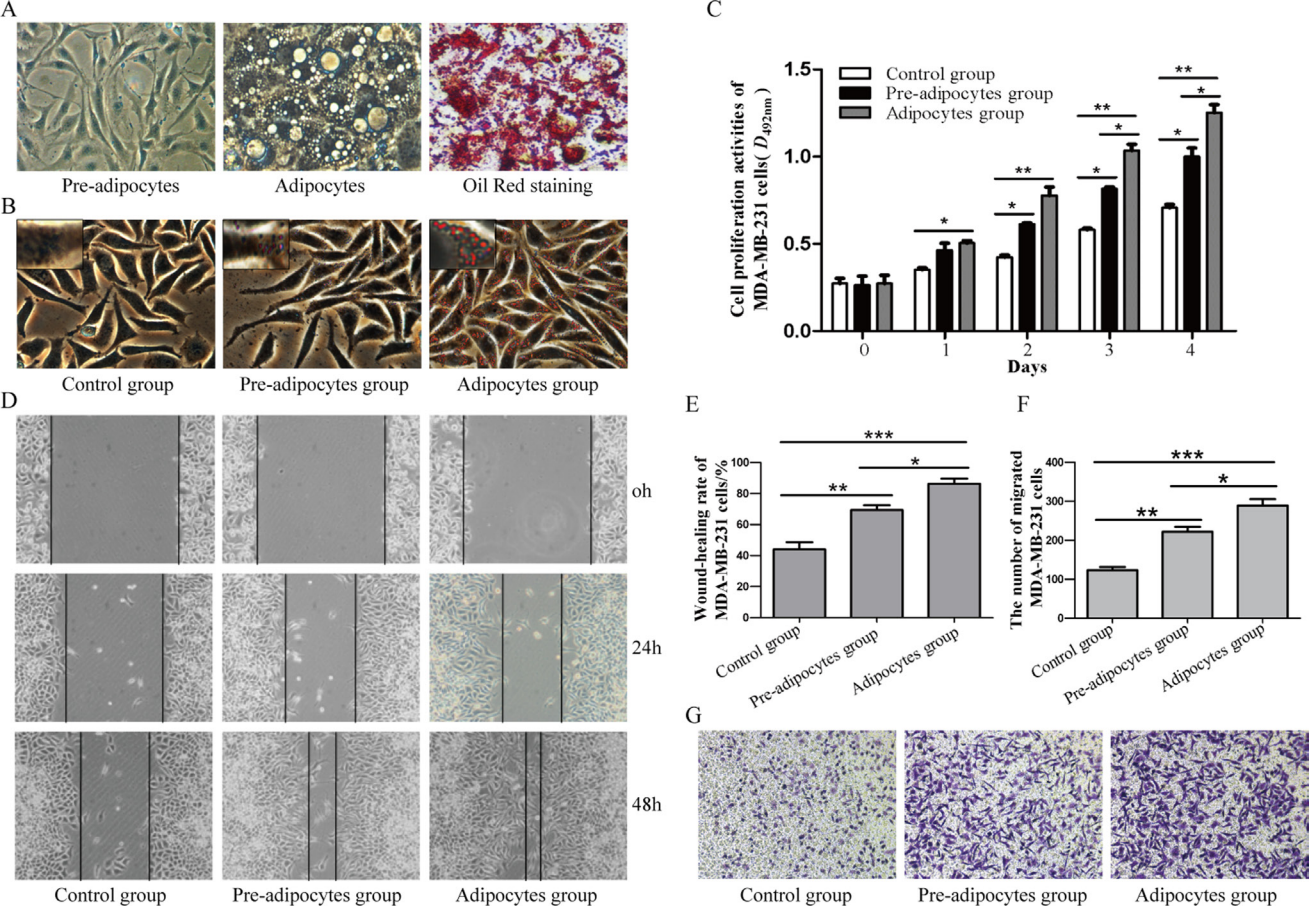
Different effects of pre-adipocytes and adipocytes on the biological behaviors of MDA-MB-231 (**A**) Adipocytic differentiation of 3T3-L1 pre-adipocytes and Oil Red staining of mature adipocytes after differentiation induction (×400). (**B**) Oil Red staining of MDA-MB-231 cells (×400). (**C**) MTT assay was performed to detect the proliferation of MDA-MB-231 cells(*P < 0.05, **P < 0.01, vs control group). Result showed that pre-adipocytes and adipocytes could promot the proliferation of MDA-MB-231 cells. (**D**) and (**E**) Cell migration was evaluated by wound-closure assay (×100; *P < 0.05, **P < 0.01, ***P < 0.001, vs control group). F and G. The migration of MDA-MB-231 cells was evaluatedby transwell migration assay (×100; *P < 0.05, **P < 0.01, ***P < 0.001, vs control group). *P < 0.05, **P < 0.01, ***P < 0.001; vs control group. Control group: MDA-MB-231; Pre-adipocytes group: MDA-MB-231+ pre-adipocytes; Adipocytes group: MDA-MB-231+ adipocytes.

There was a large amount of lipid in mature adipocytes after differentiation induction of 3T3-L1 pre-adipocytes by Oil Red staining (Figure 1A). Oil red staining of MDA-MB-231 cells showed more lipid drops in adipocytes group (Figure 1B). MTT assay showed that both pre-adipocytes and adipocytes could significantly promote the proliferation of MDA-MB-231 cells (*P <* 0.05), but this was more obvious in adipocytes group (*P <* 0.05) (Figure 1C). In addition, the wound-closure rate and the number of migrated MDA-MB-231 cells markedly increased in pre-adipocytes group and adipocytes group as compared to control group (*P <* 0.05), and the number of migrated MDA-MB-231 cells in adipocytes group was significantly higher than in pre-adipocytes group (Figure 1D–1G).

### Expression of BMP9 in MDA-MB-231 cells after co-culture

**Figure 2:**
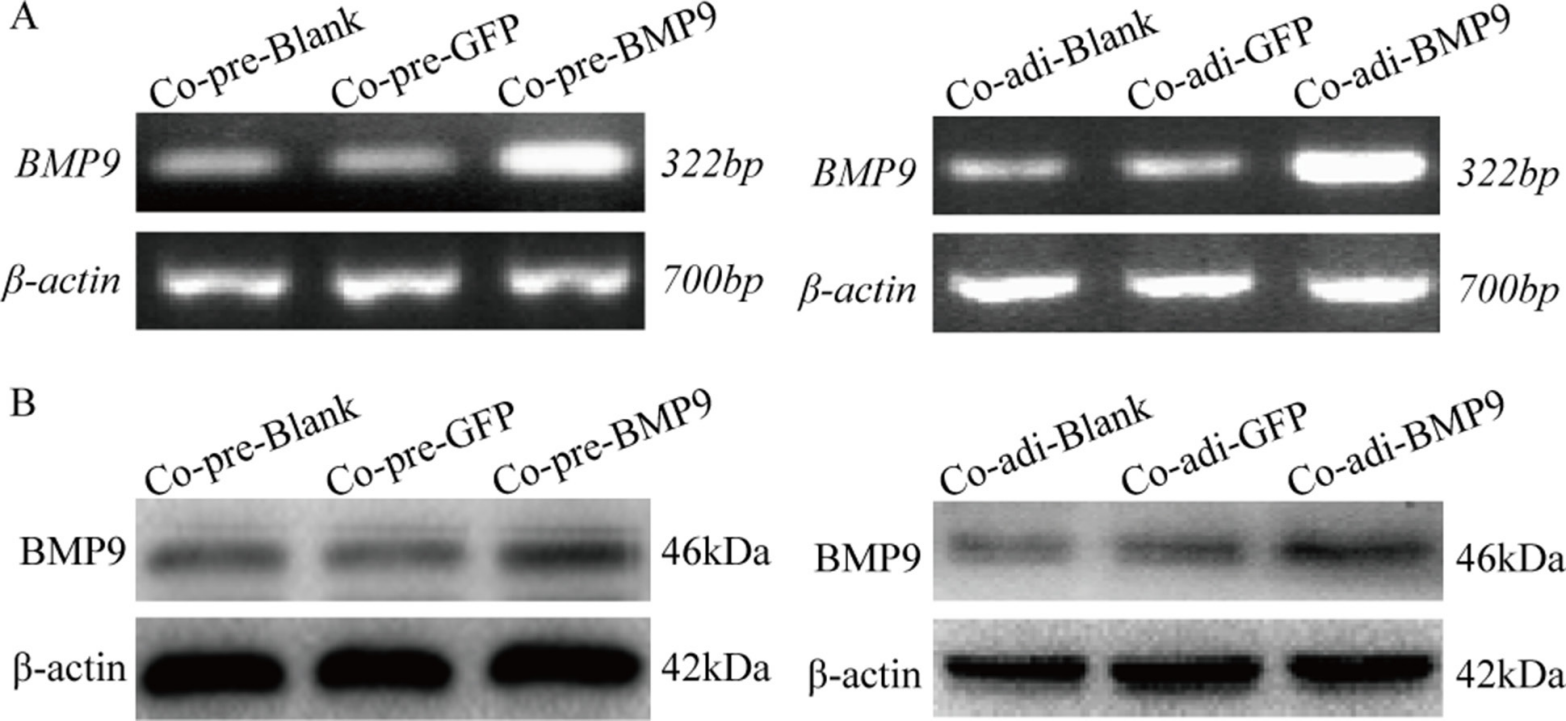
BMP9 expression in MDA-MB-231cells ofthe co-culture system was detected by RT-PCR and Western blotting (**A**) mRNA expression of BMP9 was detected by RT-PCR. (**B**) Protein expression of BMP9 was detected by Western blotting. Co-pre-Blank: MDA-MB-231 + pre-adipocytes; Co-pre-GFP: MDA-MB-231/AdGFP + pre-adipocytes; Co-pre-BMP9: MDA-MB-231/AdBMP9 + pre-adipocytes; Co-adi-Blank: MDA-MB-231 + adipocytes; Co-adi-GFP: MDA-MB-231/AdGFP + adipocytes; Co-adi-BMP9: MDA-MB-231/AdBMP9 + adipocytes.

MDA-MB-231 cells were transfected with recombinant adenovirus AdBMP9 (AdGFP as the control), and co-cultured with pre-adipocytes or adipocytes. RT-PCR (Figure 2A) and Western blotting (Figure 2B) showed that the mRNA and protein expression of BMP9 was significantly increased in MDA-MB-231 cells after transfection as compared to control group, suggesting BMP9 over-expression in these cells.

### BMP9 inhibited the proliferation and migration of MDA-MB-231 cells in the co-culture system

**Figure 3:**
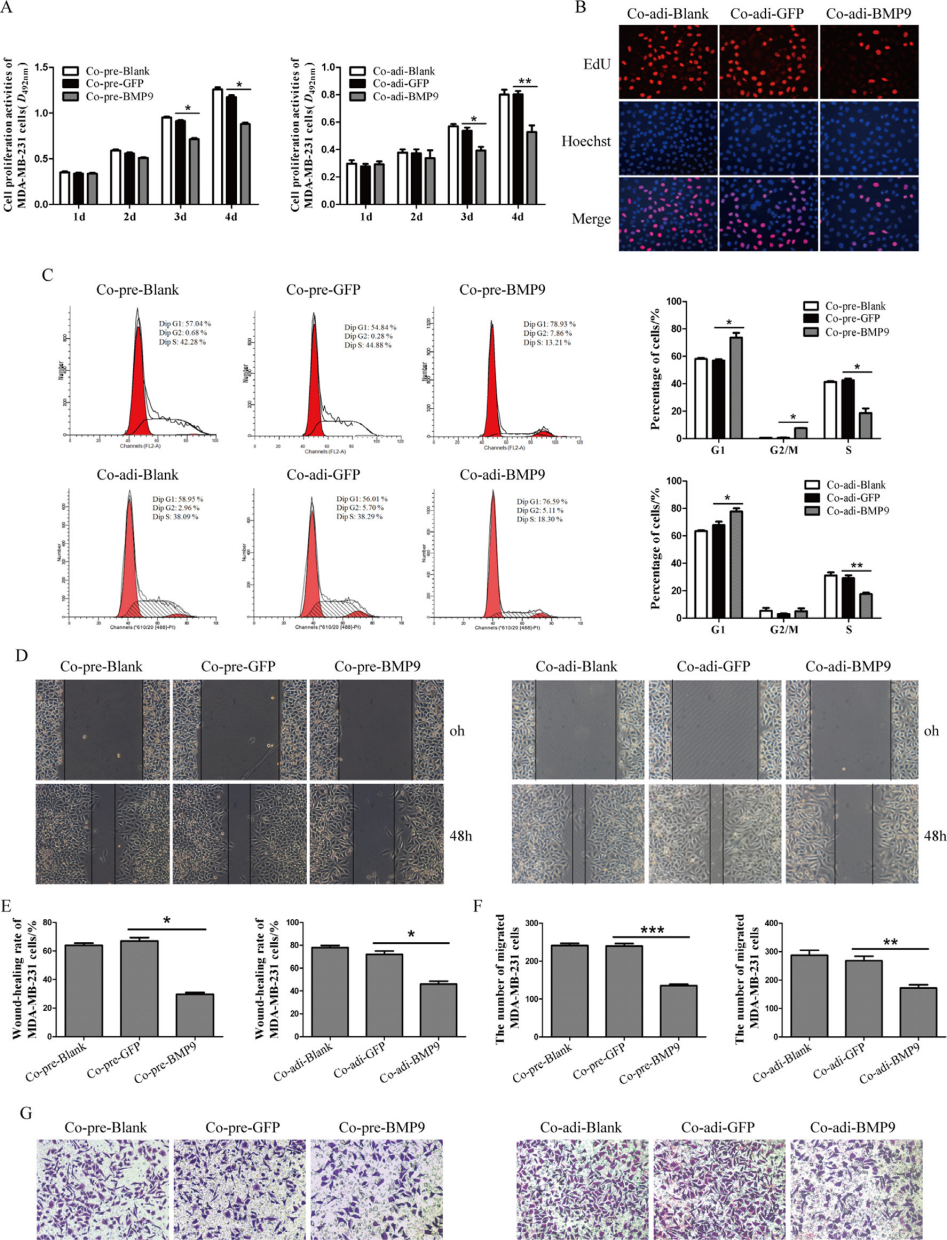
Effects of BMP9 on the proliferation and migration of MDA-MB-231 cells in the co-culture system (**A**) The proliferation of MDA-MB-231 cells in the co-cultured system was evaluated by MTT assay (*P < 0.05, **P < 0.01, vs control groups). (**B**) The proliferation of MDA-MB-231 cells in the co-cultured system was evaluated by EdU assay (×400). (**C**) The cell cycleof MDA-MB-231 cells was analyzed by flow cytometry (*P < 0.05, **P < 0.01, vs control groups). (**D**) and (**E**) The migration of MDA-MB-231 cells in the co-cultured system was evaluated by wound-closure assay (*P < 0.05, vs control group). (**F**) and (**G**) The migration of MDA-MB-231 cells in the co-culture system was evaluated by transwell migration assay (*P < 0.05, **P < 0.01, ***P < 0.001, vs control groups). All experiments were performed in triplicate.*P < 0.05, **P < 0.01, ***P < 0.001; vs control group. Results showed that BMP9 could inhibit the proliferation and migration of MDA-MB-231 cells. Co-pre-Blank: MDA-MB-231 + pre-adipocytes; Co-pre-GFP: MDA-MB-231/AdGFP + pre-adipocytes; Co-pre-BMP9: MDA-MB-231/AdBMP9 + pre-adipocytes; Co-adi-Blank: MDA-MB-231 + adipocytes; Co-adi-GFP: MDA-MB-231/AdGFP + adipocytes; Co-adi-BMP9: MDA-MB-231/AdBMP9 + adipocytes.

MTT assay showed the proliferation of MDA-MB-231 cells was markedly inhibited by BMP9 on the third day and forth day, respectively (*P <* 0.05) (Figure 3A). Similar result was shown by EdU assay on day 3 (Figure 3B). Flow cytometry showed BMP9 treatment for 72 h in the co-cultured system increased the cancer cells in G_2_/M phase (co-cultured with pre-adipocytes) and those in G_1_ phase (co-cultured with pre-adipocytes and adipocytes), but decreased cancer cells in S phase (co-cultured with pre-adipocytes and adipocytes) (*P <* 0.05) (Figure 3C). These results suggest that BMP9 suppresses the proliferation of MDA-MB-231 cells in the co-culture system.

As shown in wound-closure assay and Transwell migration assay, the wound-closure rate and the number of migrated MDA-MB-231 cells in Co-pre-BMP9 group and Co-adi-BMP9 group were markedly decreased (*P <* 0.05) as compared to Co-pre-GFP group or Co-adi-GFP group (Figure 3D–3G). These results suggest that BMP9 suppresses the migration ability of MDA-MB-231 cells in the co-culture system.

### Effect of BMP9 on the expression of leptin and Ob-R in the co-culture system

**Figure 4:**
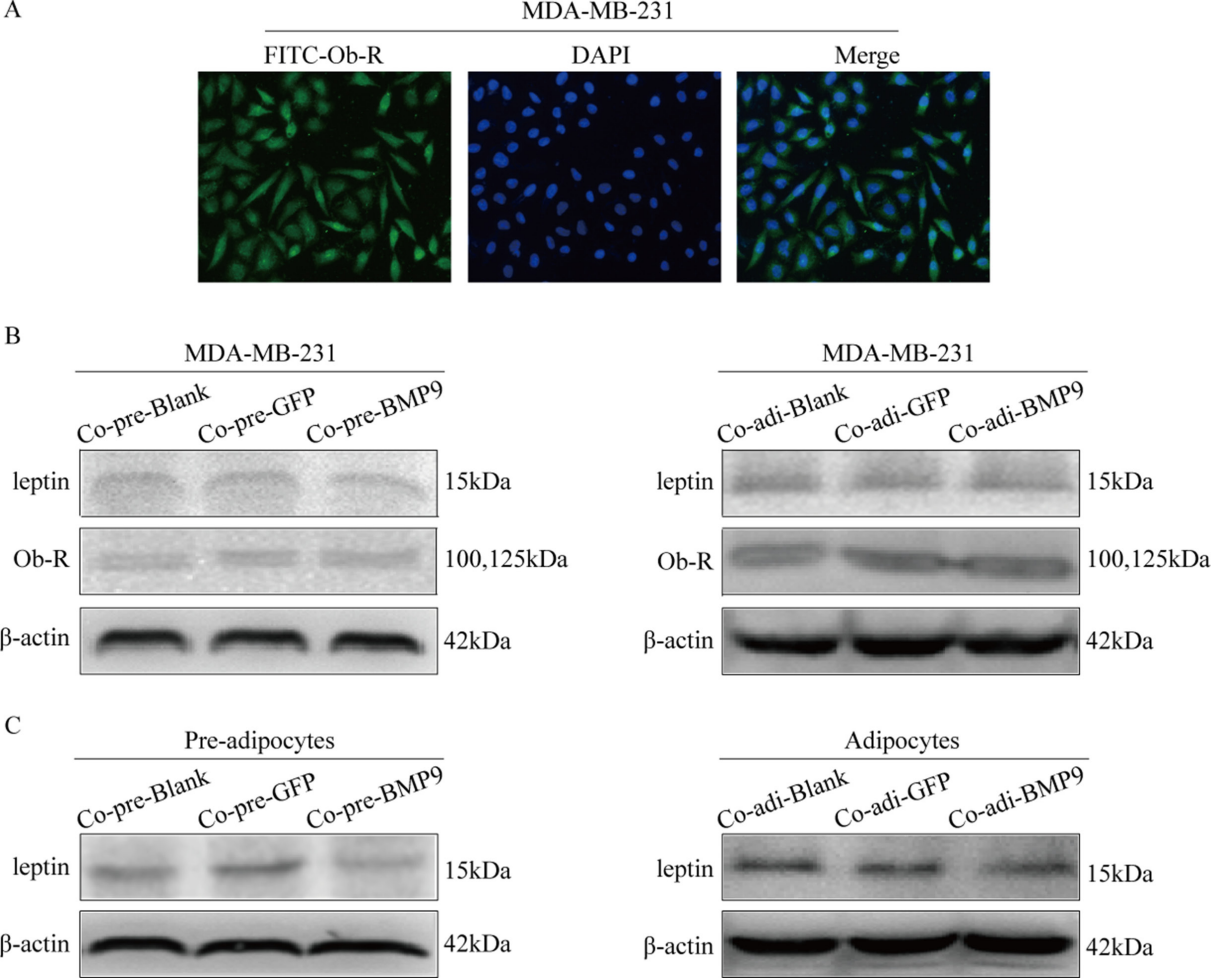
Effect of BMP9 on the expression of leptin and ObR in the co-culture system (**A**) The ObR expression in MDA-MB-231 cells was detected by immunofluorescence staining (×400). (**B**) The effect of BMP9 on the expression of leptin and ObR in MDA-MB-231 cells was detected by Western blotting. (**C**) The effect of BMP9 on the expression of leptin in pre-adipocytes or adipocytes was evaluated by Western blotting. All experiments were performed in triplicate. BMP9 had no effect on the expression of leptin and Ob-R of MDA-MB-231 cells, but inhibited the expression of leptin in the pre-adipocytes and adipocytes. Co-pre-Blank: MDA-MB-231 + pre-adipocytes; Co-pre-GFP: MDA-MB-231/AdGFP + pre-adipocytes; Co-pre-BMP9: MDA-MB-231/AdBMP9 + pre-adipocytes; Co-adi-Blank: MDA-MB-231 + adipocytes; Co-adi-GFP: MDA-MB-231/AdGFP + adipocytes; Co-adi-BMP9: MDA-MB-231/AdBMP9 + adipocytes.

The ObR expression in MDA-MB-231 cells was detected by immunofluorescence staining (Figure 4A), and the expression of leptin and ObR in pre-adipocytes and adipocytes of the co-culture system were detected by Western blotting. Notably, BMP9 significantly inhibited the leptin expression in the pre-adipocytes and adipocytes (*P <* 0.05) (Figure 4C), while it had no significant influence on the expression of leptin and Ob-R in MDA-MB-231 cells (Figure 4B).

### BMP9 inhibited the proliferation and migration of MDA-MB-231 cells by blocking leptin signaling pathway in the co-culture system

**Figure 5:**
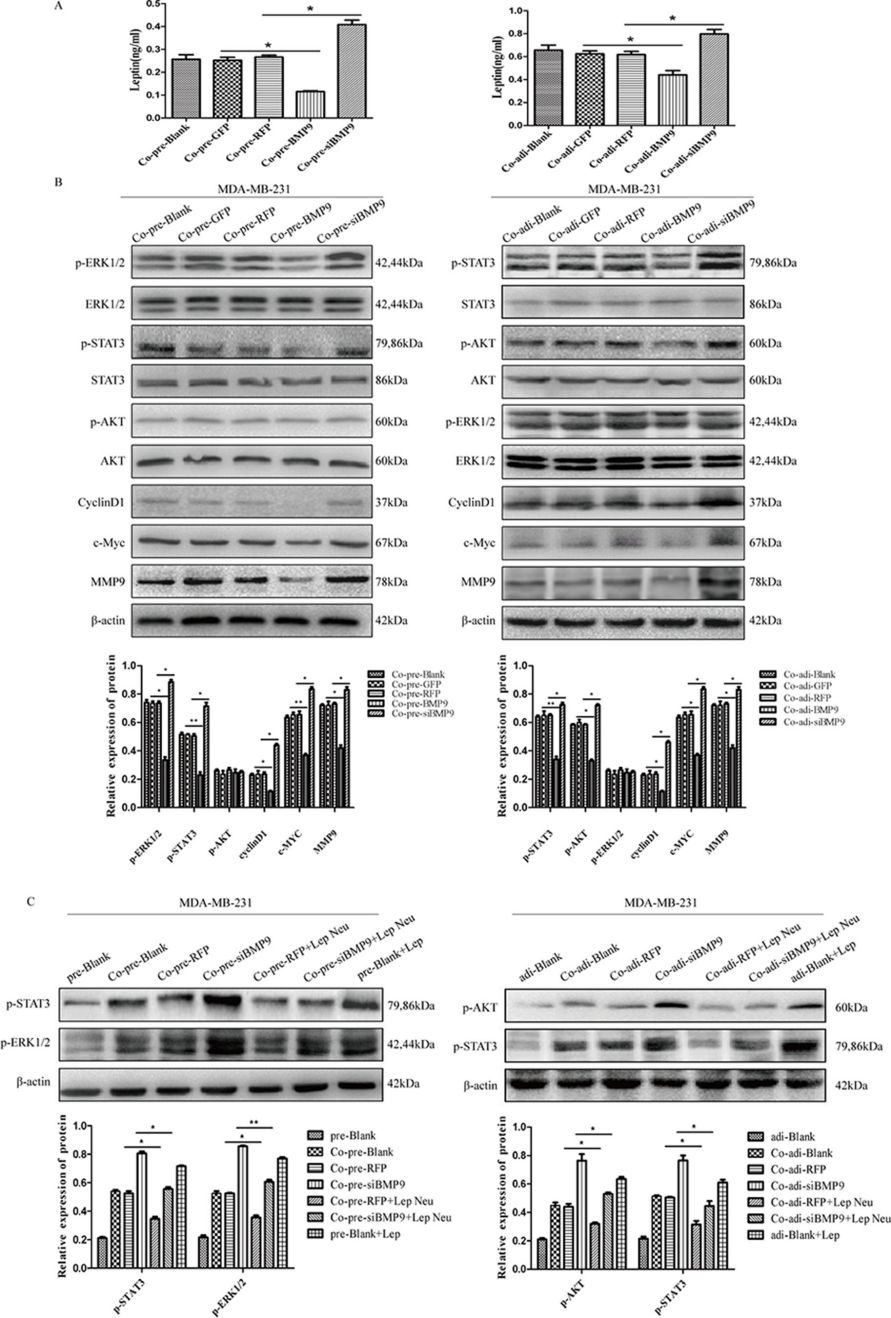
BMP9 inhibited the proliferation and migration of MDA-MB-231 cells by blocking leptin signaling pathway in the co-culture system (**A**) ELISA showed BMP9 reduced the leptin content (*P < 0.05, vs control group). (**B**) Western blotting indicated that BMP9 inhibited the activation of leptin signaling pathway (*P < 0.05, **P < 0.01, vs control groups). (**C**) Effect of leptin neutralizing antibody on the expression of p-STAT3, p-ERK1/2 and p-AKT (*P < 0.05, **P < 0.01, vs control groups). All experiments were performed in triplicate. pre-Blank: MDA-MB-231; Co-pre-Blank: MDA-MB-231 + pre-adipocytes; Co-pre-GFP: MDA-MB-231/AdGFP + pre-adipocytes; Co-pre-RFP: MDA-MB-231/AdRFP + pre-adipocytes; Co-pre-BMP9: MDA-MB-231/AdBMP9 + pre-adipocytes; Co-pre-siBMP9: MDA-MB-231/AdsiBMP9 + pre-adipocytes; Co-pre-RFP + Lep Neu: MDA-MB-231/AdRFP + pre-adipocytes + Neu-leptin; Co-pre-siBMP9 + Lep Neu: MDA-MB-231/AdsiBMP9 + pre-adipocytes + Neu-leptin; pre-Blank + Lep: MDA-MB-231 + leptin protein; adi-Blank: MDA-MB-231; Co-adi-blank: MDA-MB-231 + adipocytes; Co-adi-GFP: MDA-MB-231/AdGFP + adipocytes; Co-adi-RFP: MDA-MB-231/AdRFP + adipocytes; Co-adi-BMP9: MDA-MB-231/AdBMP9 + adipocytes; Co-adi-siBMP9: MDA-MB-231/AdsiBMP9 + adipocytes; Co-adi-RFP + Lep Neu: MDA-MB-231/AdRFP + adipocytes + Neu-leptin; Co-adi-siBMP9 + Lep Neu: MDA-MB-231/AdsiBMP9 + adipocytes + Neu-leptin; adi-Blank + Lep: MDA-MB-231 + leptin protein.

MDA-MB-231 cells were transfected with AdBMP9 (AdGFP as the control) and AdsiBMP9 (AdRFP as the control), and then co-cultured with pre-adipocytes or adipocytes. The expression of leptin in the supernatant was detected by ELISA. Results showed BMP9 over-expression reduced the leptin content, while silencing of endogenous BMP9 expression increased the leptin content (Figure 5A). Western blotting was performed to detect the molecules in the leptin signaling pathway of MDA-MB-231 cells. After co-culture with pre-adipocytes, BMP9 inhibited the expression of phosphorylated STAT3 and ERK1/2, but had no effect on the AKT expression; After co-culture with adipocytes, BMP9 inhibited the expression of phosphorylated STAT3 and AKT, but had no effect on the ERK1/2 expression in MDA-MB-231 cells. After co-culture with either pre-adipocytes or adipocytes, BMP9 reduced the protein expression of CyclinD1, c-Myc and MMP9 (Figure 5B). These results indicate that BMP9 prevent the activation of leptin signaling pathway.

Leptin neutralizing antibody was used to further verify our findings. MDA-MB-231 cells were transfected with AdsiBMP9 and co-cultured with pre-adipocytes or adipocytes. Before co-culture, leptin neutralizing antibody was added at 1 μg/ml and the medium was refreshed once every 24 h. Results showed AdsiBMP9 induced phosphorylation of STAT3 (co-cultured with pre-adipocytes and adipocytes), ERK1/2 (co-cultured with pre-adipocytes) and AKT (co-cultured with adipocytes) was reversed by leptin neutralizing antibody (Figure 5C). It confirms that leptin signaling pathway is involved in the BMP9-mediated proliferation and migration of MDA-MB-231 cells in the co-culture system.

### BMP9 inhibited the growth of MDA-MB-231 cells and reduced the expression of metastasis-related proteins *in vivo*

**Figure 6:**
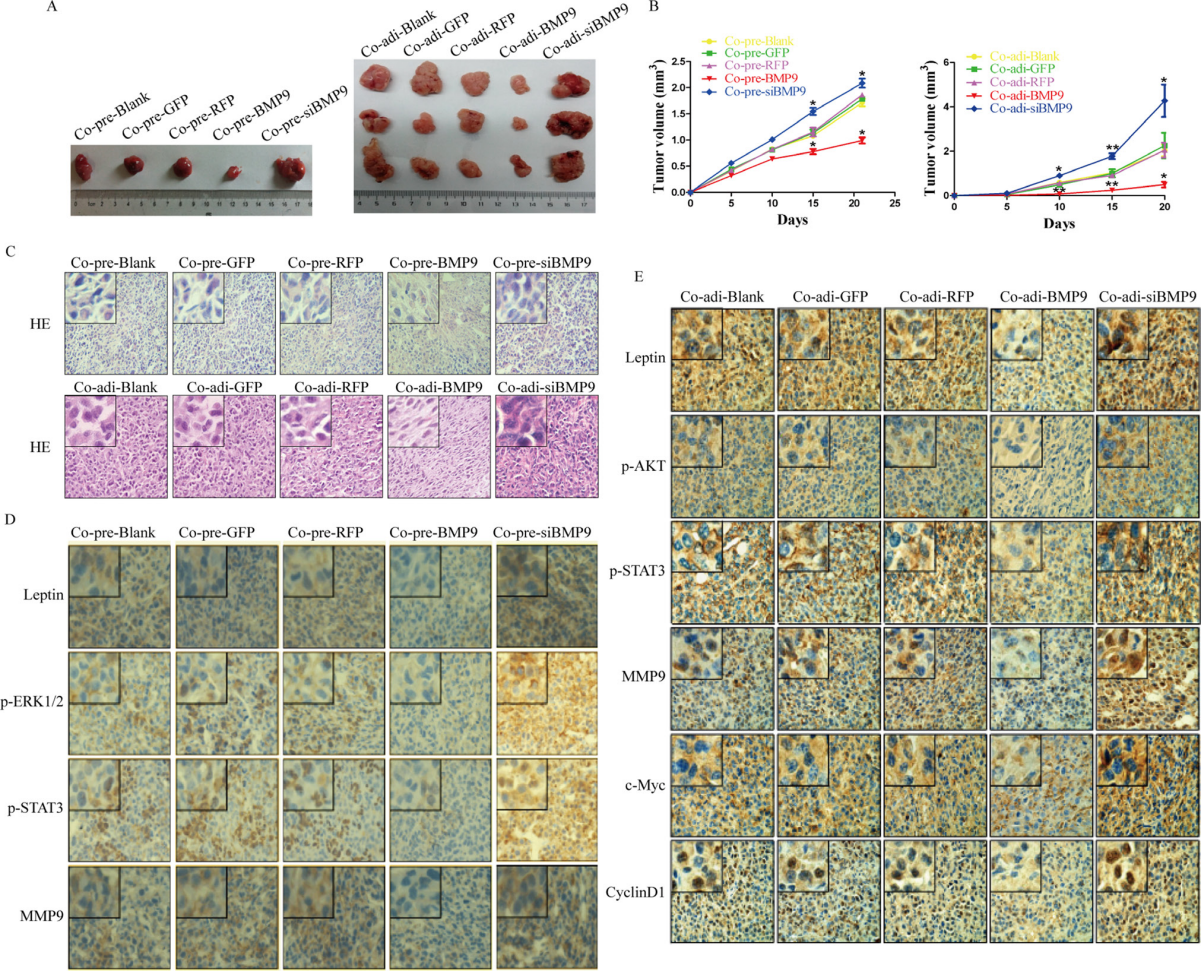
Antitumor effect of BMP9 *in vivo* (**A**) Tumors were collected from all groups. (**B**) Tumor growth curve of different groups. Results showed BMP9 inhibited the growth of MDA-MB-231 cells *in vivo*. (**C**) Hematoxylin-eosin staining of the tumors in different groups (×400). (**D**) and (**E**) Immunohistochemistry of tumors in different groups (×400). Co-pre-Blank: MDA-MB-231 + pre-adipocytes; Co-pre-GFP: MDA-MB-231/AdGFP + pre-adipocytes; Co-pre-RFP: MDA-MB-231/AdRFP + pre-adipocytes; Co-pre-BMP9: MDA-MB-231/AdBMP9 + pre-adipocytes; Co-pre-siBMP9: MDA-MB-231/AdsiBMP9 + pre-adipocytes; Co-adi-Blank: MDA-MB-231 + adipocytes; Co-adi-GFP: MDA-MB-231/AdGFP + adipocytes; Co-adi-RFP: MDA-MB-231/AdRFP + adipocytes; Co-adi-BMP9: MDA-MB-231/AdBMP9 + adipocytes; Co-adi-siBMP9: MDA-MB-231/AdsiBMP9 + adipocytes.

To further investigate the effect of BMP9 on the proliferation and migration of MDA-MB-231 cells in the co-culture system *in vivo*, pre-adipocytes or adipocytes were subcutaneously inoculated into nude mice with MDA-MB-231cells at a ratio of 5:1. Tumor volume was measured once every 5 days. Results showed tumor volume was significantly smaller on day 15 in Co-pre-BMP9 group and on day 10 in Co-adi-BMP9 group than in Co-pre-GFP group or Co-adi-GFP group (*P <* 0.05) (Figure 6A and 6B). Moreover, HE staining showed the cells in Co-pre-BMP9 group and Co-adi-BMP9 group were loosely arranged, and their nuclei were smaller than in Co-pre-GFP group and Co-adi-GFP group (Figure 6C). Immunohistochemistry (IHC) showed there were a lower expression of leptin, p-STAT3, c-Myc, CyclinD1 and MMP9 in Co-pre-BMP9 group and Co-adi-BMP9 group, a lower expression of p-ERK1/2 in Co-pre-BMP9 group and a lower expression of p-AKT in Co-adi-BMP9 group than in Co-pre-GFP group or Co-adi-GFP group. After silencing of BMP9 in Co-pre-siBMP9 group and Co-adi-siBMP9 group, the above changes were opposite to those in Co-pre-BMP9 group or Co-adi-BMP9 group (Figure 6D and 6E). These findings suggest that BMP9 suppresses the growth of xenograft tumor and inhibits the expression of leptin, p-ERK1/2, p-AKT, p-STAT3, c-Myc, CyclinD1 and MMP9 *in vivo*.

**Figure 7:**
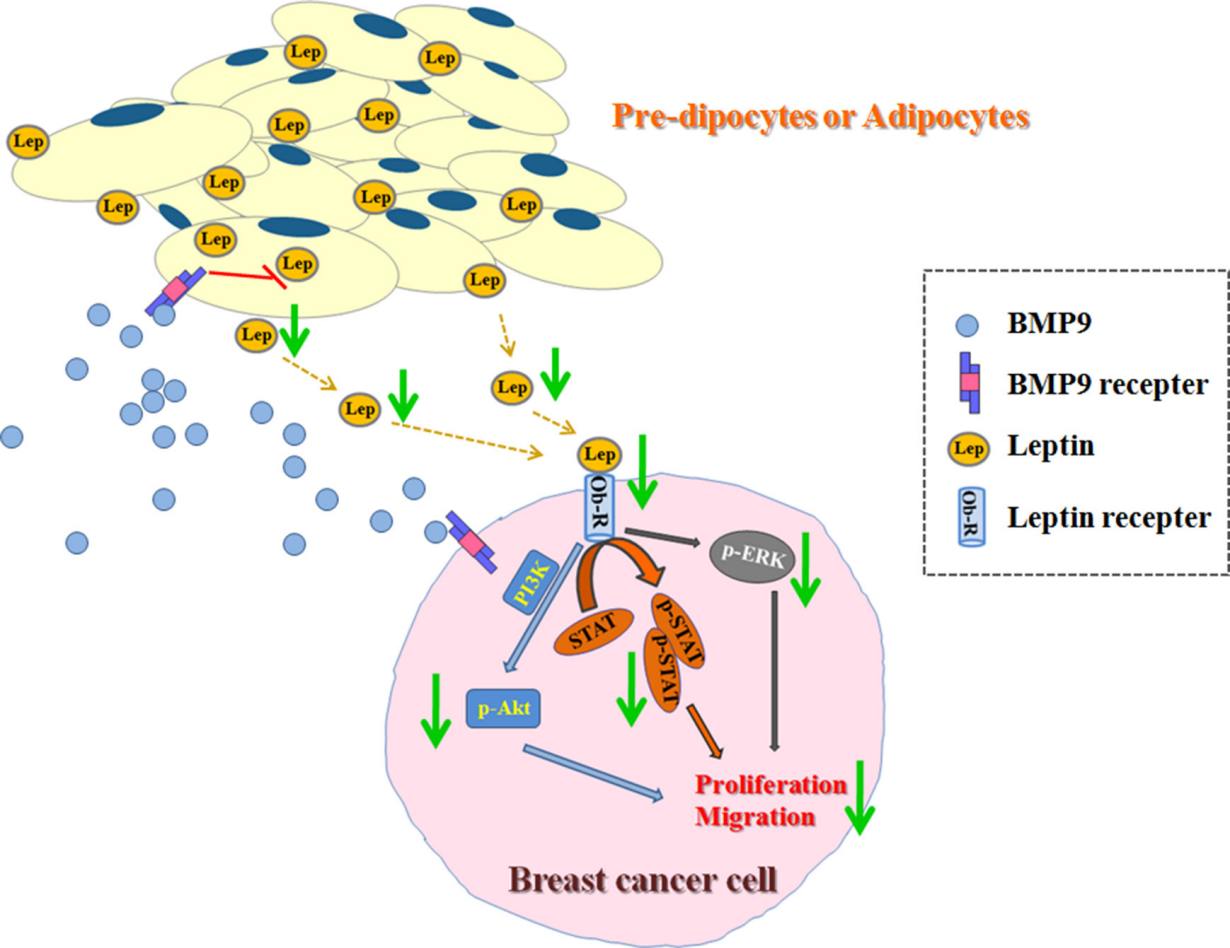
Effects of BMP9 on the biological behaviors of breast cancer cells in the co-culture system

## DISCUSSION

About 10–2 0% of breast cancer is triple-negative breast cancer (TNBC). Due to lack of endocrine and anti-HER2 therapeutic targets, TNBC often metastasize to the bone, brain and/or visceral organ in early stage, leading to a poor prognosis [26]. Recently, increasing studies focus on the relationship between obesity and cancer. Obesity may increase the risk for TNBC [27], which is, at least partially, ascribed to the influence of adipokine leptin [28]. Our previous studies demonstrated that BMP9 was able to inhibit the proliferation, migration and invasion of triple-negative MDA-MB-231 cells and HER2-positive SK-BR-3 cells, and promote their apoptosis *in vitro* and *in vivo* [23, 24]. In the present study, pre-adipocytes/adipocytes were co-cultured with MDA-MB-231 cells to mimic the adipose microenvironment, in which BMP9 was over-expressed in MDA-MB-231 cells. Our findings demonstrated that BMP9 could significantly inhibit the proliferation and migration of breast cancer cells and decrease the expression of leptin in pre-adipocytes and adipocytes of the co-culture system *in vitro* and *in vivo*. In addition, adipocytes were more potent to promote the growth of breast cancer cells than the pre-adipocytes were, but more studies are needed to prove that pre-adipocytes and adipocytes can promote the migration of breast cancer cells.

Cell proliferation and migration are crucial processes in the tumorigenesis. In this study, the effects of BMP9 on the proliferation and migration of MDA-MB-231 cells were investigated in the co-culture system. Our results showed that BMP9 over-expression inhibited the proliferation and migration of MDA-MB-231 cells, and arrested cells in G_2_/M phase (co-cultured with pre-adipocytes) and G_1_ phase (co-cultured with pre-adipocytes and adipocytes) in the co-culture system. These findings suggest that BMP9 may function as a tumor suppressor in the co-culture system. However, the underlying mechanisms are still unclear.

Leptin is one of the most important adipose-derived cytokine and mainly expressed in adipose tissues. However, recent studies indicate leptin can also be synthesized in breast cancer cells in response to obesity-related stimuli [29–31]. Leptin can bind to the ObR in MDA-MB-231 cells and then activate the STAT3, MAPK/ERK and PI3K/AKT signaling pathways, which in turn promotes the cell proliferation and migration. In our study, results showed ObR expression in MDA-MB-231 cells, and BMP9 down-regulated the leptin expression in pre-adipocytes and adipocytes, but had no effect on the leptin expression in MDA-MB-231 cells. Furthermore, AdBMP9 transfection decreased the expression of phosphorylated STAT3 (co-cultured with pre-adipocytes and adipocytes), ERK1/2 (co-cultured with pre-adipocytes) and AKT (co-cultured with adipocytes) in MDA-MB-231 cells. These results suggest that leptin signaling pathway may be involved in the BMP9 induced inhibition of proliferation and migration of MDA-MB-231 cells.

To elucidate whether leptin signaling pathway is involved in the BMP9-mediated inhibition of proliferation and migration of MDA-MB-231 cells in the co-culture system, leptin neutralizing antibody was used to block the leptin signaling pathway. Results showed that the phosphorylation of STAT3 (co-cultured with pre-adipocytes and adipocytes), ERK1/2 (co-cultured with pre-adipocytes) and AKT (co-cultured with adipocytes) was inhibited by the leptin neutralizing antibody, suggesting that BMP9 may affect cell proliferation and migration via the leptin signaling pathway.

Furthermore, the antitumor effect of BMP9 against MDA-MB-231 cells in the co-culture system was also investigated *in vivo*. Our results indicated that BMP9 inhibited the tumor growth of xenografts *in vivo*, down-regulated leptin expression, inhibited the phosphorylation of STAT3, ERK1/2, AKT, and reduced the expression of c-Myc, CyclinD1 and MMP9 in the breast cancer, while opposite results were observed in Co-pre-siBMP9 group and Co-adi-siBMP9 group. These results demonstrate that BMP9 is able to inhibit the cancer growth and inactivate the leptin signaling pathway *in vivo*.

Nude mice were sacrificed on days 20 and 21 after subcutaneous inoculation. Although significant difference was observed in tumor volume, more time is needed to evaluate the metastasis of breast cancer cells and thus intravenous inoculation of breast cancer cells is preferred for the observation of metastasis.

In conclusion, our finding show that BMP9 is able to inhibit the proliferation and migration of breast cancer in adipose microenvironment. The expression of growth and metastasis related factors (leptin, cyclinD1, c-Myc and MMP9) is decreased by BMP9. Leptin signaling pathway is involved in the anti-tumor effects of BMP9 in adipose microenvironment. These results are also confirmed *in vivo*. Thus, our study suggests that BMP9 may be used as a novel therapy for breast cancer.

## MATERIALS AND METHODS

### Cell culture and recombinant adenoviruses

Human breast cancer cell line MDA-MB-231 cells and mouse pre-adipocyte cell line 3T3-L1 cells were purchased from the China Center for Type Culture Collection (CCTCC) and American Type Culture Collection (ATCC), respectively. MDA-MB-231 cells were maintained in Dulbecco's modified Eagle's medium (DMEM) supplemented with 10% fetal bovine serum (FBS; Gibco, USA), 100 U/ml penicillin and 100 μg/ml streptomycin, while 3T3-L1 cells were maintained in DMEM supplemented with 10% new born calf serum (NBCS; Gibco, USA), 100 U/ml penicillin and 100 μg/ml streptomycin, at 37°C in a humidified atmosphere containing 5% CO_2_.

Recombinant adenovirus expressing BMP9 (AdBMP9) and adenovirus expressing green fluorescent protein (AdGFP) were kindly provided by Dr. Tong-chuan He in the University of Chicago Medical Center, USA. Recombinant adenovirus interfering BMP9 (AdsiBMP9) and negative control adenovirus (AdRFP) expressing red fluorescent protein were generated previously using the AdEasy system. All recombinant adenoviruses were amplified in HEK293 (CCTCC) cells before use [32].

### Differentiation of 3T3-L1 cells into mature adipocytes

3T3-L1 pre-adipocytes were cultured until 100% confluence. Then, they were induced to differentiate into mature adipocytes with induction medium (0.5 mmol/L methylisobutylxanthine, 1 μmol/ml dexamethasone, and 1 μg/ml insulin, and 10% FBS) for 2 days, with 2 μg/ml insulin for another 2 days, and finally with regular DMEM containing 10% FBS for additional 4 days.

### Oil red staining

Adipocytes were washed twice with phosphate buffered saline (PBS; pH 7.4), and then fixed with 4% paraformaldehyde for 30 min. 1.5 ml Oil Red staining solution was added to each well, followed by incubation for 30 min. After washing twice with PBS, cells were observed under a light microscope.

### Indirect co-culture

3T3-L1 pre-adipocytes or adipocytes were co-cultured with MDA-MB-231 cells using a Transwell culture system (0.4 μm pore size; Corning). MDA-MB-231 cells (1.5 × 10^5^/well) and 3T3-L1 pre-adipocytes or adipocytes (2 × 10^4^/chamber) were seeded into 6-well culture plates and culture chamber, respectively. After 12-h culture, MDA-MB-231 cells were transfected with AdBMP9, AdGFP, AdsiBMP9 or AdRFP. Eight hours later, the medium was refreshed with DMEM containing 1% FBS. Then, the inserts were transferred into six-well plates, followed by co-culture.

### Cell proliferation assay

MTT assay was performed in quintuplicate to assess the viability of MDA-MB-231 cells in the co-culture system. After co-culture with 3T3-L1 pre-adipocytes or adipocytes, 500 μl of MTT solution (5 mg/ml; Progema, Madison, WI, USA) was added into each well, followed by incubation for another 4 h. Then, 3.75 ml of dimethyl sulfoxide was added to each well. The absorbance of each well was measured once daily for consecutive 4 days at 492 nm using a microplate reader, and a growth curve was delineated. Experiment was repeated three times.

### Flow cytometry

In brief, MDA-MB-231 cells were collected after 3-day co-culture. Cells were washed twice with ice-cold PBS (pH 7.4) and re-suspended. Cell cycle analysis was done using a Xow cytometer and the CellQuest software package. Experiment was performed at least thrice.

### Wound-closure assay

A wound-closure assay was conducted to assess the migration of MDA-MB-231 cells. When the cell confluence reached 80%, a wound was made in the center of the cell monolayer with a 10-μl sterile pipette tip. After co-culture, cell migration was observed by a light microscope, and images were captured at 0, 24 and 48 h after the wound was made. The wound-healing rate was calculated as follow: (width at 0 h – width at 48 h)/width at 0 h × 100%. The experiment was performed thrice.

### Transwell migration assay

After co-culture for 2 days with 3T3-L1 pre-adipocutes or adipocytes, MDA-MB-231 cells were subjected to migration assay. Approximately 5 × 10^4^ MDA-MB-231 cells were suspended in 0.4 ml of serum-free medium from the co-culture system. Then, the cell suspension was added to the upper chamber, and the medium from co-culture system supplemented with 10% FBS was added to the lower chamber as a chemoattractant. Twenty-four hours later, the migrated cells were washed twice with PBS, fixed in 4% paraformaldehyde and stained with crystal violet for 30 min. The migrated cells were counted under a light microscope at ×100 magnification. Means were obtained from five randomly selected fields in each well. The experiment was repeated three times.

### RNA isolation and RT-PCR

Total RNA was extracted from MDA-MB-231 in the co-culture system using Trizol reagent (Invitrogen, Carlsbad, CA, USA) according to the manufacturer's instructions. After reverse transcription, cDNA was amplified with 1 μg of total RNA using a Primer Script Kit (TaKaRa, Dalian, China). The mRNA expression of target genes was normalized to that of β-actin. Primers used in this study are shown in Table 1.

**Table 1:** Sequences of primers

Genes		Sequences	Product length (bp)
β-actin	Forward	5′-CACCACACCTTCTACAATGAGC-3′	700
	Reverse	5′-GTGATCTCCTTCTGCATCCTGT-3′	
BMP9	Forward	5′-CTGCCCTTCTTTGTTGTCTT-3′	322
	Reverse	5′-CCTTACACTCGTAGGCTTCATA-3′	

### Immunofluorescence staining

MDA-MB-231 cells in 24-well cell plate were washed twice with PBS and fixed with 4% paraformaldehyde for 30 min. Immunofluorescence staining was performed using Ob-R antibody (Santa Cruz, sc-8391, 1:200). Images were captured under a fluorescence microscope at 400×.

### Western blotting

After co-culture for 3 days, proteins were extracted from MDA-MB-231 cells, pre-adipocytes and adipocytes. The protein concentration was determined by bicinchoninic acid assay. After denaturation in boiling water, proteins were separated by 10% SDS-polyacrylamide gel electrophoresis and then transferred onto PVDF membranes. The membranes were blocked with 5% bovine serum albumin at 37°C for 2 h, incubated with a primary antibody at 4°C overnight and then with a corresponding secondary antibody (ABGENT, ASS1007, ASS1009, 1:1000). After washing with TBST, the target protein on the membranes was visualized with an enhanced chemiluminescence substrate kit (Millipore Corporation, Billerica, MA, USA). The primary antibodies used in this study were anti-β-actin (Santa Cruz Biotechnology, sc-47778, 1:1000), anti-BMP9 (Abcam, ab71809, 1:200), anti-Leptin (Abcam, ab3583, 1:500), anti-Ob-R (Santa Cruz Biotechnology, sc-8391, 1:1000), anti-STAT3 (CST, #4904, 1:2000), anti-p-STAT3 (CST,# 9145, 1:2000), anti-AKT (CST, #4691, 1:1000), anti-p-AKT (CST, #3375, 1:1000), anti-ERK1/2 (CST, #4695, 1:1000), anti-p-ERK1/2 (CST, #4370, 1:2000), anti-c-Myc(Santa Cruz Biotechnology, sc-40, 1:1000), anti-CyclinD1 (Santa Cruz Biotechnology, sc-753, 1:1000) and anti-MMP9 (Abcam, ab137867, 1:2000). The expression of proteins was normalized to that of β-actin. Data were analyzed using the Bio-Rad Electrophoresis Documentation (Gel Doc 1000) and Quantity One version 4.5.0 (Bio-Rad, Hercules, CA, USA).

### *In vivo* tumorigenesis assay

All experiments were approved by the Institutional Animal Care and Use Committee of the Chongqing Medical University. Four-week-old female nude mice (Balb/c, Beijing, China) were housed under specific pathogen-free condition. MDA-MB-231 cells were transfected with AdGFP, AdRFP, AdBMP9 or AdsiBMP9 *in vitro*. Pre-adipocytes (2.5 × 10^7^ cells) or adipocytes (2.5 × 10^7^ cells) and MDA-MB-231 cells (5 × 10^6^ cells) were inoculated subcutaneously into female nude mice in different groups. Five days later, tumors were observable. Tumor volume was measured once every 5 days and tumor growth curve was delineated. Nude mice were sacrificed 20 days later, tumor tissues were collected, paraffin-embedded, and stained with hematoxylin-eosin.

### Immunohistochemistry

Paraffin-embedded tumor tissues were sectioned. The sections were deparaffinized and rehydrated. After antigen retrieval in citric acid buffer, sections were treated with 3% hydrogen peroxide to block the activity of endogenous catalase, and then incubated with the primary antibody at 4°C overnight. After incubation with secondary antibody for 20 min, sections were visualized with 3, 3-diaminobenzidine tetrachloride. Finally, sections were counterstained with hematoxylin, and mounted.

### Statistical analysis

All the data are presented as mean ± standard deviation and analyzed with SPSS 17.0. Student's *t* test was used to determine the significant differences between two groups. A value of *P* < 0.05 was considered statistically significant.

## Abbreviations

ObR: leptin receptor
BMPs: bone morphogenetic proteins
TGF-β: transforming growth factor-β
EMT: epithelial-mesenchymal transition
IHC: immunohistochemistry
TNBC: triple-negative breast cancer
CCTCC: China Center for Type Culture Collection
ATCC: American Type Culture Collection
DMEM: Dulbecco's modified Eagle's medium
FBS: fetal bovine serum
NBCS: new born calf serum
AdBMP9: adenovirus expressing BMP9
AdGFP: adenovirus expressing green fluorescent protein
AdsiBMP9: adenovirus interfering BMP9
AdRFP: negative control adenovirus
